# Burden of Aging: Health Outcomes Among Adolescents and Young Adults With Sickle Cell Disease

**DOI:** 10.1097/HS9.0000000000000930

**Published:** 2023-07-13

**Authors:** Kristen E. Howell, Norma Pugh, Jennifer Longoria, Nirmish Shah, Abdullah Kutlar, Victor R. Gordeuk, Allison A. King, Jeffrey Glassberg, Mariam Kayle, Cathy Melvin, Marsha Treadwell, Jane S. Hankins, Jerlym S. Porter

**Affiliations:** 1 Department of Epidemiology and Cancer Control, St. Jude Children’s Research Hospital, Memphis, TN, USA; 2 RTI International, Research Triangle Park, NC, USA; 3 Department of Psychology, St. Jude Children’s Research Hospital, Memphis, TN, USA; 4 Department of Medicine, Duke University, Durham, NC, USA; 5 Department of Medicine, Augusta University, Augusta, GA, USA; 6 University of Illinois at Chicago, IL, USA; 7 Washington University School of Medicine, St. Louis, MO, USA; 8 Department of Emergency Medicine, Icahn School of Medicine at Mount Sinai, New York, NY, USA; 9 Clinical Health Systems and Analytics Division, Duke University, Durham, NC, USA; 10 Department of Public Health Sciences, Medical University of South Carolina, Charleston, SC, USA; 11 Department of Pediatrics, University of California San Francisco Benioff Children’s Hospital Oakland, San Francisco, CA, USA; 12 Department of Hematology, St. Jude Children’s Research Hospital, Memphis, TN, USA

Although ≈95% of children with sickle cell disease (SCD) will reach adulthood in high-income settings,^1–4^ adolescents and young adults (AYA) face difficulties in establishing adult care and experience progression of disease severity as they age.^5–7^ As patients leave pediatric care, they undergo life events as emerging adults such as graduating high school, attending college, or joining the work force. Along with the progression of their disease severity, these life changes introduce stressors impacting their mental health and psychosocial functioning, but are not well characterized. We aimed to identify differences in health-related outcomes (ie, clinical and psychosocial), and transition barriers between adolescents (age, 15–17 years) and young adults (YAs) (age, 18–25 years) to help inform the burden of aging with SCD. We hypothesized that YAs with SCD experience increased severity of health-related outcomes (ie, increased clinical outcomes and decreased psychosocial functioning) and increased transition barriers compared with adolescents with SCD.

This analysis was conducted as part of the Sickle Cell Disease Implementation Consortium (SCDIC), a cooperative research program aimed at using implementation science research to accelerate the translation of evidence-based therapies into clinical care among individuals with SCD ages 15–45 years through research studies and a longitudinal registry.^8^ The current study included AYA aged 15–25 years with SCD, enrolled in the SCDIC registry.^9,10^ As previously described,^11^ baseline data were gathered from 2016 to 2019. This study was approved by the institutional review board and written informed consent was obtained from all participants or their legal guardian if the participant was a minor.

Demographics included gender, age, SCD genotype, insurance type, race, ethnicity, primary language, marital status, number of children and adults living in the household, household income, education, and occupation. Other covariates included age at SCD diagnosis, transfusion history, pain history, hydroxyurea utilization, and type of healthcare professional providing the majority of SCD care in the past 2 years. Participants were stratified by age at the baseline assessment to compare adolescents (age, 15.0–17.9 years) and YAs (age, 18.0–25.0 years).

Clinical outcomes included records of common SCD-related dysfunctional organs: joint osteonecrosis, chronic kidney disease, stroke, hypertension, skin ulcers, retinopathy, and chronic refractory pain. Clinical outcomes were extracted from the medical records using standardized definitions^10^ and summarized to reflect the total number of clinical outcomes ever experienced since study enrollment (0, 1, and ≥2 outcomes). No weights were given to different outcomes, as a consensus of severity scores in SCD is lacking and infrequently accounts for the patient’s experience.

Healthcare resource utilization was extracted from the medical records to reflect the total number of visits in the past 12 months, including acute pain/infusion center, emergency department, and hospitalizations. Barriers to receiving medical care in the past 12 months included 11 items about concerns about cost, insurance, timing, transportation, severity of the complications, previous poor experiences, and language barriers. Barriers were summarized to reflect the total number of barriers experienced in the past 12 months.

Psychosocial factors were measured by the National Institutes of Health resource HealthMeasures, which includes 4 validated health-related quality of life measurement systems.^12^ The systems used in this study were the Adult Sickle Cell Quality of Life Measurement Information System (ASCQ-Me),^13^ Quality of Life in Neurological Disorders (Neuro-QoL),^14^ and Patient-Reported Outcomes Measurement Information System (PROMIS).^15^ Item responses for the ASCQ-Me and Neuro-QOL outcomes were uploaded to the HealthMeasures Scoring Service, where T-scores were generated. The ASCQ-Me^13^ assessed sleep impact and reliance on others. Higher T-scores indicated more desirable outcomes (ie, better sleep and less reliance on others).^13^ The Neuro-QOL^14^ assessed cognitive functioning and task management. Higher T-scores indicated more desirable outcomes (ie, better cognitive function and better task management).^14^ Depression was measured using the 4-item PROMIS^15^ short form for Emotional Distress-Depression. Higher T-scores indicated less desirable outcomes (ie, more severe depression).

The aim was to identify differences in 10 health-related outcomes between adolescents and YAs. Univariate models examined the relationships between each outcome and the covariates. All covariates statistically significant at *P* < 0.1 were included as candidate variables for the final multivariable models. To prevent collinearity, variables significantly correlated with age group, the primary covariate of interest, were not included in the model. Backward elimination, using a significance cutoff of 0.05, was used to identify the best fitting models, and age group was included in each multivariable model, regardless of significance. Analyses were conducted in SAS Version 9.4 (SAS Institute Inc., Cary, NC).

A total of 996 SCDIC registry participants met inclusion criteria. Baseline characteristics of the adolescents (n = 214; 21.5%) and YAs (n = 782; 78.5%) are presented in Table 1. YAs were more likely to be high school graduates, employed, receive regular blood transfusions, have severe pain, have pain so bad that it was hard to finish tasks, less likely to see a SCD specialist or hematologist for most of their care, and more likely to have taken hydroxyurea compared with adolescents.

**Table 1:** Study Characteristics

Characteristic	Young Adults (N=782)	Adolescents (N=214)	*P*-Value*
**Demographics**			
**Age (years**)^**W**^, Mean (std)	21.7 (2.3)	15.9 (0.8)	**<.001**
**Gender^C^**, N (%)			
Male	352 (45.0%)	106 (49.5%)	.240
Female	430 (55.0%)	108 (50.5%)	
**Genotype^C^**, N (%)			
Non-severe Sickling: SC/S Beta+/S-HPFH	204 (26.1%)	65 (30.4%)	.211
Severe Sickling: SS/S Beta 0/SD/SO/SE	578 (73.9%)	149 (69.6%)	
Insurance type*^C^, N (%)			
Private	239 (31.9%)	64 (30.5%)	.065
Public	468 (62.5%)	142 (67.6%)	
None	42 (5.6%)	4 (1.9%)	
**Marital Status**			
Married or living as married	29 (4.1%)	0 (0.0%)	.391
Other	677 (95.9%)	35 (100.0%)	
**Household income^C^**, N (%)			
$25,000 or less	353 (53.0%)	85 (47.2%)	.169
$25,001+	313 (47.0%)	95 (52.8%)	
**Education^C^**, N (%)			
Less than high school graduate	87 (11.3%)	194 (94.6%)	**<.001**
High school graduate or higher	684 (88.7%)	11 (5.4%)	
Employment*^C^, N (%)			
Engaged	209 (28.7%)	19 (9.3%)	**<.001**
Unengaged	520 (71.3%)	185 (90.7%)	
**Other Covariates**			
**Regular blood transfusions^C^**, N (%)			
Yes	211 (27.3%)	40 (19.0%)	**.014**
No	563 (72.7%)	171 (81.0%)	
**Frequency of very severe pain (past 6 mo)^C^**, N (%)			
Never	101 (13.0%)	42 (20.2%)	**<.001**
Rarely	163 (21.0%)	68 (32.7%)	
Sometimes	263 (33.9%)	69 (33.2%)	
Often	213 (27.4%)	26 (12.5%)	
Always	36 (4.6%)	3 (1.4%)	
**What type of healthcare professional has been providing the majority of care for your sickle cell disease in the past 2 years^C^**, N (%)			
Sickle cell specialist or hematologist	595 (87.4%)	181 (94.3%)	**.039**
Primary care or general practice	41 (6.0%)	7 (3.6%)	
Emergency department	34 (5.0%)	4 (2.1%)	
I don’t currently receive care for my sickle cell disease	11 (1.6%)	0 (0.0%)	
**Have you ever taken hydroxyurea^C^**, N (%)			
Yes	529 (74.0%)	136 (66.0%)	**.025**
No	186 (26.0%)	70 (34.0%)	
**Clinical Outcomes**			
No. of dysfunctional organs*^C^, N (%)			
0	376 (48.1%)	149 (69.6%)	**<.001**
1	260 (33.2%)	52 (24.3%)	
2+	146 (18.7%)	13 (6.1%)	
**No. visits in the past year for acute pain/crisis**^N^, N	665	190	
Mean (std)	5.0 (8.8)	2.2 (2.6)	**<.001**
**Mental Health**			
Depression T-score*^W^, N	771	209	
Mean (std)	46.1 (8.1)	43.7 (7.0)	**<.001**
**Depression treatment^C^**, N (%)			
Currently receiving treatment	60 (7.9%)	16 (7.6%)	**.006**
Treated in the past but not now	100 (13.1%)	11 (5.2%)	
Never received treatment	602 (79.0%)	183 (87.1%)	
**Anxiety (Medical Abstraction Form)^C^**, N (%)			
Yes	96 (19.4%)	19 (10.8%)	**.009**
No	399 (80.6%)	157 (89.2%)	
**Functioning**			
Sleep Impact T-score*^W^, N	772	208	
Mean (std)	50.3 (10.0)	53.8 (8.7)	**<.001**
Cognitive Functioning T-score*^W^, N	772	209	
Mean (std)	49.2 (9.5)	47.8 (9.0)	**.041**
Task Management T-score*^W^, N	778	210	
Mean (std)	53.2 (8.2)	51.7 (8.7)	**.024**
Reliance on others T-score*^W^, N	775	210	
Mean (std)	52.2 (9.9)	54.7 (8.9)	**.002**
**Barriers to Medical Care**			
**No. barriers summed 0-12 in the last 12M**^**N**^, N	782	214	
Mean (std)	0.6 (1.3)	0.1 (0.4)	**<.001**

C = Chi-square test; F = Fisher’s Exact test; HPFH = hereditary persistence of fetal hemoglobin; N = negative binomial test; SC = compound heterozygous for hemoglobin S and hemoglobin C; SD = compound heterozygous for hemoglobin S and hemoglobin D; SE = compound heterozygous for hemoglobin S and hemoglobin E; SO = compound heterozygous for hemoglobin S and hemoglobin O; SS = homozygous for hemoglobin S; W = Wilcoxon Rank Sum test.

* Participants with both private and public insurance are categorized as ‘private’. ‘Engaged’ employment includes participants who are students and/or employed. ‘Unengaged’ employment includes participants who are unemployed and/or disabled. Dysfunctional organs include: Avascular Necrosis, Chronic kidney disease, Stroke, Pulmonary arterial hypertension, skin ulcers, retinopathy, and Chronic refractory pain. For Depression T-Score: higher scores indicate less desirable outcomes (i.e., more severe depression). For Functioning T-scores (Sleep Impact, Cognitive Functioning, Task Management, Reliance on others): higher scores indicate more desirable outcomes (i.e., better sleep, less reliance on others).

Bold text is used to identify *P*-values less than 0.05.

At the time of study enrollment, 51.9% of YAs and 30.4% of adolescents had experienced at least 1 event of organ dysfunction (Table 1). YAs having a severe SCD genotype, receiving regular blood transfusions, and higher pain frequency were associated with more dysfunctional organs (Table 2). Compared with adolescents, YAs experienced significantly more avascular necrosis, stroke, pulmonary hypertension, retinopathy, and chronic pain (Suppl. Table S1).

**Table 2:** Multivariable model for clinical and psychosocial outcomes

Outcome: Dysfunctional Organs Covariate	Odds ratio (95% CI) of ‘2+ dysfunctional organs’	*P*-value*
Age group (YA vs. A)	**2.57 (1.778, 3.760**)	**<.001**
Genotype (Severe vs. Less Severe)	**1.51 (1.070, 2.128**)	**.020**
Regular blood transfusions (Yes vs. No)	**1.87 (1.330, 2.637**)	**<.001**
How often have very severe pain		**.009**
Never	1.00 (Reference)	1.00
Rarely	1.14 (0.694, 1.892)	.609
Sometimes	**1.81 (1.134, 2.909**)	**.014**
Often	**1.96 (1.193, 3.247**)	**.009**
Always	**2.60 (1.038, 6.500**)	**.035**
Majority of care		**.005**
Not currently receiving care	1.00 (Reference)	1.00
SCD specialist	5.58 (0.928, 106.851)	.122
PCP	1.80 (0.258, 36.464)	.615
Emergency department	2.92 (0.420, 59.003)	.359
**Outcome: Number of acute pain/crisis visits in past 12 months**		
**Covariate**	**Point Estimate**	***P*-value**
Age group (YA vs. A)	**2.58 (1.290, 3.878**)	**<.001**
Hydroxyurea (Ever vs. Never)	**2.29 (1.079, 3.511**)	**<.001**
**Outcome: Barriers to medical care in the past 12 months**		
**Covariate**	**Point Estimate**	***P*-value**
Age group (YA vs. A)	**0.48 (0.297, 0.669**)	**<.001**
Gender (Male vs. Female)	**-0.32 (-0.470, -0.161**)	**<.001**
Majority of care		
Not currently receiving care	0.00 (Reference)	1.00
SCD specialist	-0.17 (-0.857, 0.525)	.638
PCP	-0.05 (-0.805, 0.714)	.907
Emergency department	0.77 (-0.004, 1.551)	.051
**Outcome: Depression** **Covariate**	**Point Estimate**	***P*-value**
Age group (YA vs. A)	**1.75 (0.459, 3.033**)	**.008**
Income (>$25K vs ≤$25K)	-1.04 (-2.091, 0.003)	.051
How often have very severe pain		
Never	0.00 (Reference)	1.00
Rarely	1.36 (-0.371, 3.099)	.123
Sometimes	**3.14 (1.517, 4.761**)	**<.001**
Often	**5.84 (4.086, 7.596**)	**<.001**
Always	**8.22 (5.261, 11.176**)	**<.001**
**Outcome: Depression Treatment** **Covariate**	**Odds ratio (95% CI) of ‘current treatment’**	***P*-value**
Age group (YA vs. A)	1.35 (0.835, 2.262)	.229
Gender (Male vs. Female)	**0.65 (0.442, 0.9941**)	**.024**
Insurance		
None	1.00 (Reference)	1.00
Private	1.02 (0.415, 2.882)	.974
Public	1.71 (0.738, 4.662)	.255
Ethnicity (Hispanic vs. Non-Hispanic)	**2.39 (0.937, 6.179**)	**.022**
How often have very severe pain		
Never	1.00 (Reference)	1.00
Rarely	1.49 (0.699, 3.394)	.320
Sometimes	**2.32 (1.174, 5.026**)	**.022**
Often	**3.75 (1.877, 8.212**)	**<.001**
Always	**3.94 (1.387, 11.131**)	**.008**
**Outcome: Anxiety** **Covariate**	**Odds ratio (95% CI) of ‘Anxiety’**	***P*-value**
Age group (YA vs. A)	7.07 (1.452, 127.564)	.058
Regular blood transfusions (Yes vs. No)	**2.26 (1.362, 3.740**)	**.002**
Hydroxyurea (Yes vs. No)	**2.19 (1.184, 4.340**)	**.017**
**Outcome: Sleep Impact** **Covariate**	**Point Estimate**	***P*-value**
Age group (YA vs. A)	**-1.96 (-3.403, -0.516**)	**<.001**
Gender (Male vs. Female)	**1.36 (0.188, 2.529**)	**.008**
How often have very severe pain		
Never	0.00 (Reference)	1.00
Rarely	**-2.97 (-4.898, -1.048**)	**.003**
Sometimes	**-5.90 (-7.719, -4.088**)	**<.001**
Often	**-8.98 (-10.929, -7.041**)	**<.001**
Always	**-11.32 (-14.603, -8.040**)	**<.001**
**Outcome: Cognitive Functioning** **Covariate**	**Point Estimate**	***P*-value**
Age group (YA vs. A)	**1.99 (0.561, 3.450**)	**.007**
How often have very severe pain		
Never	0.00 (Reference)	1.00
Rarely	**-2.33 (-4.277, -0.383**)	**.019**
Sometimes	**-4.10 (-5.935, -2.263**)	**<.001**
Often	**-4.55 (-6.502, -2.591**)	**<.001**
Always	**-6.79 (-10.098, -3.474**)	**<.001**
**Outcome: Task Management** **Covariate**	**Point Estimate**	***P*-value**
Age group (YA vs. A)	**1.46 (0.175, 2.737**)	**<.001**
**Outcome: Reliance on Others** **Covariate**	**Point Estimate**	***P*-value**
Age group (YA vs. A)	-0.54 (-1.882, 0.803)	.431
How often have very severe pain		
Never	0.00 (Reference)	1.000
Rarely	**-4.07 (-5.868, -2.272**)	**<.001**
Sometimes	**-8.08 (-9.781, -6.389**)	**<.001**
Often	**-12.55 (-14.347, -10.743**)	**<.001**
Always	**-16.61 (-19.671, -13.552**)	**<.001**

A = adolescents; CI = confidence interval; PCP = primary care provider; SCD = sickle cell disease; YA = young adults.

* Bold text is used to identify *P*-values less than 0.05.

The mean number of acute visits over the past 12 months was 5.0 (±8.8) for YAs and 2.2 (±2.6) for adolescents (Table 1). YAs and those with prior use of hydroxyurea had significantly more acute visits over the past 12 months (Table 2). The mean number of barriers to medical care was 0.6 (±1.3) among YAs and 0.1 (±0.4) among adolescents (Table 1). YAs and females experienced more barriers to medical care (Table 2).

The mean depression t-score was 46.1 (±8.1) among YAs and 43.7 (±7.0) among adolescents (Table 1). YAs and those with more frequent pain were associated with higher self-reported depression (Table 2). As reported by participants, 79.0% of YAs and 87.1% of adolescents had never received treatment for depression (Table 1). Current depression treatment was significantly associated with females, Hispanic ethnicity, and pain frequency (Table 2). Age group was not significantly associated with current depression treatment. According to the medical record, 19.4% of YAs and 10.8% of adolescents had record of anxiety (Table 1). Anxiety was significantly associated with regular blood transfusions and prior use of hydroxyurea (Table 2). Age was borderline significantly associated with anxiety.

The mean sleep impact score was 50.3 (±10.0) among YAs and 53.8 ± (8.7) among adolescents (Table 1). YAs, females, and higher pain frequency were associated with worse sleep (Table 2). The mean self-reported cognitive functioning score was higher among YAs (49.2 [±9.5]) compared with adolescents (47.8 [±9.0]) (Table 1). Younger age and increased pain frequency were associated with worse cognition (Table 2). The mean task management score was 53.2 (±8.2) among YAs and 51.7 (±8.7) among adolescents (Table 1). YAs had significantly better task management skills (Table 2). The mean reliance score was 52.2 (±9.9) among YAs and 54.7 (±8.9) among adolescents (Table 1). More frequent pain was associated with more reliance on others (Table 2).

AYAs with SCD are a vulnerable population due to the increasing SCD severity. As hypothesized, the current study found that YAs with SCD experience more dysfunctional organs, increased acute visits, increased medical barriers, depression, and poorer sleep compared with adolescents with SCD. On the contrary, YAs reported higher cognitive function and task management than adolescents.

It is well known that the frequency of acute events and SCD-related mortality increases as patients age.^2,16–18^ The current study expanded prior work demonstrating that YAs were more likely to experience dysfunctional organs and mental health complications than adolescents, supporting and possibly explaining the rising mortality rates in young adulthood. Transition programs must anticipate the increased frequency of clinical outcomes (ie, increased disease burden) that starts in adolescence and into adulthood, thus preparing emerging adults to remain vigilant and aware of their progressive symptoms. We found that YAs were more likely to be treated with hydroxyurea, contrasting with a previous study where only 37% of YAs with SCD were prescribed hydroxyurea and prescription fills decreased as individuals aged.^19^ This difference is likely attributable to differences in study design, as population-level data that included community clinics may reflect lower access to disease-modifying therapies than our current study, which primarily comprises academic institutions.^20^

Previous research has found that among AYA with SCD, patients with elevated distress/depression reported significantly higher pain frequency than those with minimal distress/depression.^21,22^ Poor sleep has also been linked to worsened depression.^22,23^ The current study confirmed these associations between pain frequency, depression, and sleep. Although the current study found that YAs have an increased prevalence of depression, there was no difference in the treatment of depression between adolescents and YAs. This demonstrates the importance of allocating mental health resources during healthcare transitions to monitor AYAs and provide interventions to prevent added distress from life changes.

Although the adjusted association between age and anxiety was insignificant, it is important to consider how anxiety might increase during transition. During transition, AYAs often shift to an unfamiliar care environment^6^ and face financial/insurance and time-constraint barriers. The current study showed that YAs face more barriers to receiving medical care than adolescents and are less likely to receive care from an SCD specialist. Addressing anxiety and barriers to care throughout transition is important.

Strengths of this study include that it is a large sample of SCD AYA, providing sufficient power to detect age differences. Limitations of this study include that some outcomes are reported at enrollment, where participants are asked to recall events in the past year, which may introduce recall bias. Additionally, individuals potentially sought care at facilities other than the included sites; therefore, health care utilization may not be completely ascertained. Finally, the nature of the study limits our ability to explore any of causal association between outcomes and covariates.

As individuals with SCD transition to adulthood, it is crucial to anticipate the increased severity of health outcomes and have heightened attention to mental health. This study provides evidence to inform future guideline development, research investigation, and health services planning. Specifically, the AYA period requires interventions, such as (1) allocating resources toward mental health services, (2) addressing anxieties and barriers with transition programming, (3) building self-management skills to ensure patients remain engaged with their care, and (4) addressing the high frequency of pain interference and severity in YAs. These interventions must be implemented as an integral part of transition programming and continued throughout adult care.

## ACKNOWLEDGEMENTS

The authors would like to thank the members of the Sickle Cell Disease Implementation Consortium.

Jason Hodges, PhD, MA

Yvonne Carroll, RN, JD

Lisa Klesges, PhD, MS

Hamda Khan, MA

Matthew Smeltzer, PhD, MS

Chinonyelum Nwosu, MPH

James Gurney, PhD

Nicole Alberts, PhD

Reginald French

Sherif Badawy, MD, MS, MBBCh

Michael DeBaun, MD, MPH

Guolian Kang, PhD

Jeremie Estepp, MD

Winfred Wang, MD

Curtis Owens, MD

Margaret Debon, PhD

Ray Osarogiagbon, MD

Marquita Nelson, MD

Elliott Vichinsky, MD

Ted Wun, MD

Michael Potter, MD

Danielle Hessler, PhD

Ward Hagar, MD

Anne Marsh, MD

Lynne Neumayr, MD

Julie Kanter, MD

Shannon Phillips, PhD, RN

Robert Adams, MD

Martina Mueller, PhD

Paula Tanabe, PhD, MSN

Hayden Bosworth, PhD

George Jackson, PhD

Fred Johnson, MBA

Rachel Richesson, PhD

Janet Prvu-Bettger, ScD

Ana Baumann, PhD

Cecilia Calhoun, PhD

Robert Gibson, PhD

Angie Snyder, PhD

Maria Fernandez, PhD

Richard Lottenberg, MD

Lynne D. Richardson, MD

Jena Simon, MS, APRN-BC

Nicholas G. Genes, MD, PhD

George T. Loo, DrPH

Jason S. Shapiro, MD, MA

Kimberly Souffront PhD, FNP-BC, RN

Cindy Clesca, MA

Elizabeth Linton, MPH

Gery Ryan PhD, MA

Barbara L Kroner, PhD

Lucia Rojas-Smith, DrPH

Tabitha Hendershot, BA

Lisa DiMartino, PhD, MPH

Sara Jacobs, PhD

Whitney Battestilli, BA

Donald Brambilla, PhD

Sharon M Smith, PhD

William P. Tonkins, Dr. PH, J.D.

Marlene Peters-Lawrence, BSN, RN

Cheryl Boyce, PhD

Whitney Barfield, PhD

Alexis Thompson, MD

Melissa Gutierrez, MS

Jana Hirschtick, PhD

Lewis Hsu, MD, PhD

Jerry Krishnan, MD, PhD

Nadew Sebro, MD

Larissa Verda, MD, PhD

Abe Wandersman, PhD

Michael Berbaum, PhD

Kishore Bobba, MD

Joe Colla, MD

Kim Erwin, MDes

Andrea Lamont, PhD

Molly Martin, MD. MAPP

Sarah Norell, MDes, MFA

Ananta Pandit, MD

Kay Saving, MD

Robin Shannon, DNP, RN

Robert Winn, MD

Leslie Zun, MD

## AUTHOR CONTRIBUTIONS

KEH, JSH, and JSP conceptualized and designed the study, drafted the initial article, and reviewed and revised the article. NP performed data analysis, drafted the initial article, and reviewed and revised the article. JL, NS, AK, VRG, AAK, JG, MK, CM, and MT reviewed and revised the article. All authors approved the final article as submitted and agree to be accountable for all aspects of the work.

## DATA AVAILABILITY

Data availability statement: Data will be publicly available from the NHLBI Data Repository at https://biolincc.nhlbi.nih.gov/home/ starting July 2023. Until that time, please contact byk@rti.org for the original data.

## DISCLOSURES

JSH receives consultancy fees from Global Blood Therapeutics, CVS Health and Forma Therapeutics during the conduct of this study. JSP received consultancy fees from Forma Therapeutics and funding from the National Heart Lung and Blood Institute K01 HL125495 during the conduct of this study. All the other authors have no conflicts of interest to disclose.

## SOURCES OF FUNDING

The Sickle Cell Disease Implementation Consortium has been supported by US Federal Government cooperative agreements U24HL133948, U01HL133964, U01HL133990, U01HL133996, U01HL133994, U01HL133997, U01HL134004, U01HL134007, and U01HL134042 from the National Heart Lung and Blood Institute and the National Institute on Minority Health and Health Disparities (Bethesda, MD).

## Supplementary Material


