# Supplementary material for: Burden of Aging: Health Outcomes Among Adolescents and Young Adults With Sickle Cell Disease
Source: Hemasphere. 2023 Jul 13;7(8):e930. doi: 10.1097/HS9.0000000000000930 (PMC10348722; doi:10.1097/HS9.0000000000000930)
Supplement: Supplementary file 1 [file hs9-7-e930-s001.docx]

**Supplementary Material**

**Supplemental Table 1.** Organ dysfunction types by age group

| **Organ Dysfunction** | **Adolescents (N=214)** | **Young Adults (N=782)** | **Total (N=996)** | **P-Value** |
| --- | --- | --- | --- | --- |
| **Avascular necrosis^C^** |  |  |  |  |
| Yes | 22 (13.4%) | 154 (28.3%) | 176 (24.8%) | **.0001** |
| No | 142 (86.6%) | 391 (71.7%) | 533 (75.2%) |  |
| **Chronic kidney disease^C^** |  |  |  |  |
| Yes | 6 (5.8%) | 39 (8.4%) | 45 (7.9%) | .3684 |
| No | 98 (94.2%) | 425 (91.6%) | 523 (92.1%) |  |
| **Stroke^C^** |  |  |  |  |
| Yes | 19 (10.9%) | 125 (22.1%) | 144 (19.5%) | **.0010** |
| No | 156 (89.1%) | 440 (77.9%) | 596 (80.5%) |  |
| **Pulmonary hypertension^C^** |  |  |  |  |
| Yes | 6 (5.3%) | 63 (13.3%) | 69 (11.8%) | **.0179** |
| No | 107 (94.7%) | 411 (86.7%) | 518 (88.2%) |  |
| **Skin ulcers^C^** |  |  |  |  |
| Yes | 2 (1.5%) | 12 (2.6%) | 14 (2.4%) | .4551 |
| No | 130 (98.5%) | 442 (97.4%) | 572 (97.6%) |  |
| **Retinopathy^C^** |  |  |  |  |
| Yes | 18 (10.3%) | 99 (18.1%) | 117 (16.2%) | **.0150** |
| No | 157 (89.7%) | 449 (81.9%) | 606 (83.8%) |  |
| **Chronic pain^C^** |  |  |  |  |
| Yes | 10 (10.4%) | 105 (24.8%) | 115 (22.2%) | **.0022** |
| No | 86 (89.6%) | 318 (75.2%) | 404 (77.8%) |  |

C=Chi-square test

Avascular necrosis included: hip, shoulder, and knee.

Stroke included: ischemic, hemorrhagic, transient ischemic attack, and silent

Pulmonary hypertension included: mean arterial pressure ≥ 25mmHg, and tricuspid regurgitation velocity ≥ 3.0 m/s
